# Hybrid Assistive Limb Intervention for Hemiplegic Shoulder Dysfunction Due to Stroke

**DOI:** 10.7759/cureus.19827

**Published:** 2021-11-23

**Authors:** Masakazu Taketomi, Yukiyo Shimizu, Hideki Kadone, Yasushi Hada, Masashi Yamazaki

**Affiliations:** 1 Doctoral Program in Clinical Science Graduate School of Comprehensive Human Science, University of Tsukuba, Tsukuba, JPN; 2 Department of Rehabilitation Medicine, Faculty of Medicine, University of Tsukuba, Tsukuba, JPN; 3 Center for Innovative Medicine and Engineering, University of Tsukuba Hospital, Tsukuba, JPN; 4 Department of Orthopaedic Surgery, Faculty of Medicine, University of Tsukuba, Tsukuba, JPN

**Keywords:** hybrid assistive limb (hal), shoulder, robotic rehabilitation, upper limb impairment, rehabilitation, stroke

## Abstract

Upper limb dysfunction after stroke is one of the most serious functional disorders, and adequate functional recovery is often not expected. Although various studies have been conducted on effective rehabilitation for upper limb dysfunction, active rehabilitation such as repetitive training of upper limb elevation has not been sufficiently conducted yet because the shoulder joint is highly unstable and the appearance of pain is easily observed. In this study, we performed right shoulder joint elevation training in a seated position using a single-joint hybrid assistive limb (HAL) in a 54-year-old female with right hemiplegia after a stroke. Her right upper limb function improved as follows: passive and active range of motion (ROM) of shoulder flexion, from 105° to 115° and from 65° to 105°, respectively; manual muscle test (MMT), from 2 to 4; box and block test of the right hand, from 1 to 8; right grip strength, from less than 5 to 7.4 kg; and action research arm test (ARAT) total scores, from 10 to 20. No adverse events including shoulder pain were seen. According to the result of the pilot study, HAL may be an effective rehabilitation tool for upper limb dysfunction after stroke.

## Introduction

Upper limb dysfunction caused by stroke significantly affects activities of daily living (ADLs) and reduces the quality of life (QOL). It has been reported that the motor function of paralyzed limb tends to improve within six weeks, paralysis is unlikely to recover regardless of the severity three months after the onset of the stroke, and approximately 60% of patients with severe or complete paralysis are unable to perform any activities with their involved hand after six months [1,2].

Regarding upper limb dysfunction after stroke, muscle weakness in the paralyzed limb can decrease the stability of the shoulder joint, easily causing subluxation and shoulder pain that reduced the patient's QOL; therefore, training is performed focusing on protection rather than active functional training [3,4]. Once shoulder pain develops, it is difficult to improve; moreover, it hinders rehabilitation and adversely affects ADLs [5,6]. The risk of shoulder pain has been pointed out in constraint-induced therapy (CI therapy), which is now widely used in rehabilitation for upper limb dysfunction, and careful training of the paralyzed shoulder joint is still necessary [7]. However, excessive rest can cause contractures related to ADLs and pain. Adequate rehabilitation for shoulder dysfunction will be able to enhance patients with stroke.

There are various training methods focusing on upper limb functions besides shoulder function [8,9]. Robot-based rehabilitation has been attracting attention because it can reduce the burden on caregivers and ensure a sufficient amount of rehabilitation [10]. The current research on robotic rehabilitation aims at reaching and hand movements [10,11].

Hybrid assistive limb (HAL) is an exoskeleton robot that can control and assist movements based on bioelectrical potentials associated with voluntary movements. Repeated voluntary movements give the central nervous system feedback to promote functional recovery and induce plasticity in the impaired central nervous system [12]. There are various types of HAL: the lower limb type, the single-joint type, and the lumbar type. Previous research using the single-joint HAL for the elbow reported on the improvement of the upper limb motor function in patients with stroke [13].

Focusing on the shoulder dysfunction after stroke, we considered the clinical application of shoulder type HAL, which was developed in our facility [14,15] for patients after stroke. This is the first case report of active repetitive upper limb-raising training using a shoulder joint HAL for a hemiplegic patient with moderate upper limb dysfunction due to a chronic stroke.

## Case presentation

Participant

**Figure 1:**
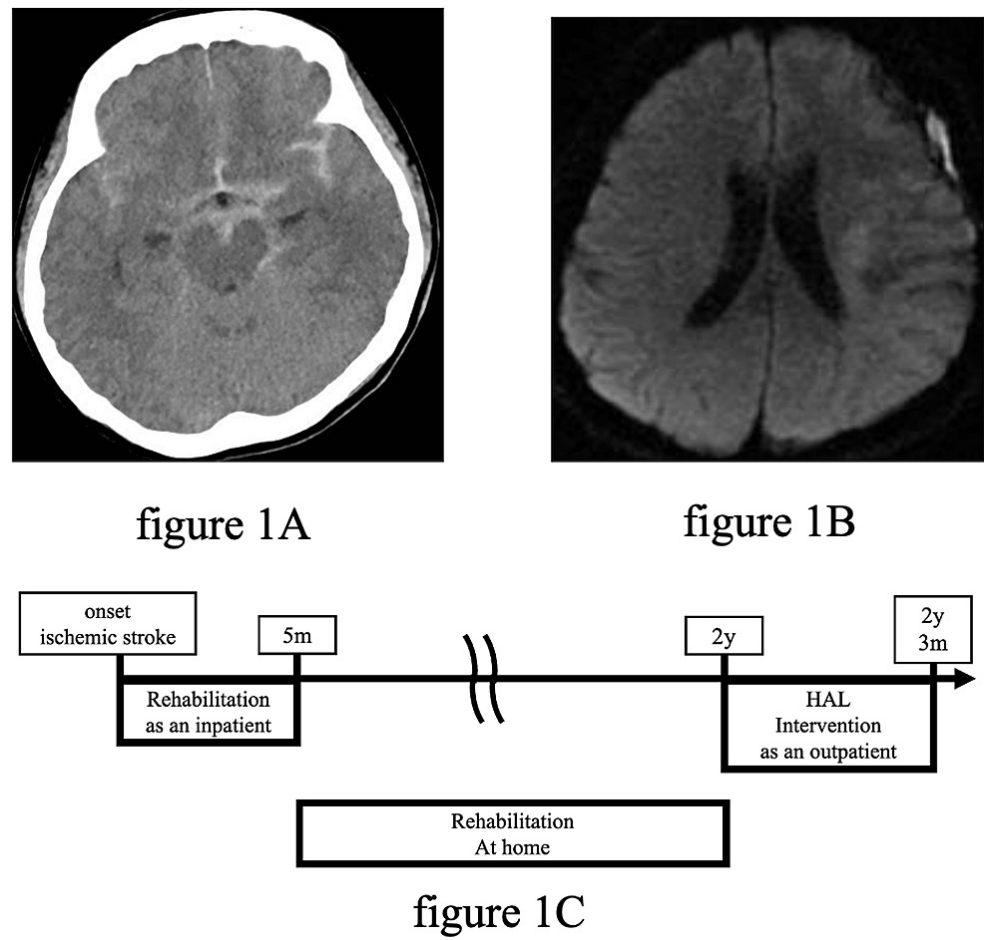
(A) A head computed tomography showing diffuse digital subtraction angiography. (B) Axial diffusion-weighted image shows a high-signal area in the corona radiata. (C) Participant's history.

A 54-year-old female with right hemiplegia presented to our hospital two years after the onset of the paralysis. She had developed a subarachnoid hemorrhage, followed by a spasm and then cerebral infarction in the left middle cerebral artery territory (Figures 1A, 1B, 1C). She had no history of hospitalization or surgeries besides this stroke episode. The patient was treated in the acute and convalescent wards, received rehabilitation training, and was discharged home five months after the onset. The rehabilitation included the exchange of the dominant hand during hospitalization, and the patient's right upper limb did not participate in daily activities at discharge. After that, she continued her rehabilitation program at home, but her right upper limb dysfunction did not improve. She continued to use the left upper limb for daily activities and rarely used the right upper limb. Two years after the onset of the disease, the patient visited our hospital with right upper limb dysfunction. Thus, we decided on the clinical application of shoulder HAL. This study was conducted in accordance with the Declaration of Helsinki, with approval from the Ethics Committee of the Tsukuba University Faculty of Medicine (approval no.: TCRB18-38). The participant provided written informed consent for participation and publication, including the use of accompanying images.

HAL intervention

Shoulder HAL

Shoulder HAL was developed using single-joint type HAL. It was originally developed for the knee and elbow joints, supporting uniaxial movements at a single joint, such as flexion and extension of the joint [14]. In order to support shoulder flexion and extension movements, the proximal arm of the robot was affixed to the platform and the distal arm of the robot to the upper arm.

We used a belt for the elbow joint that was included with the robot to attach the distal part of the HAL to the upper arm. To align the robot with the shoulder, the patient sat in an adjustable chair, and the height and position of the robot were adjusted such that the center of the rotation of the HAL was located 2-3 cm below the acromion, and the longitudinal axis of the HAL was aligned with the center of the shoulder joint. The elbow was extended to the maximum extent using a splint, and the forearm was fixed in the midline position. Sensors were placed on the surface of the anterior part of the deltoid muscle to obtain electrical output during shoulder flexion. We were cautious that the patient raised her upper limb in the scapular plane where the interference between the scapula and humerus was the least and at the angle with the least burden according to the condition of the patient's shoulder.

**Figure 2:**
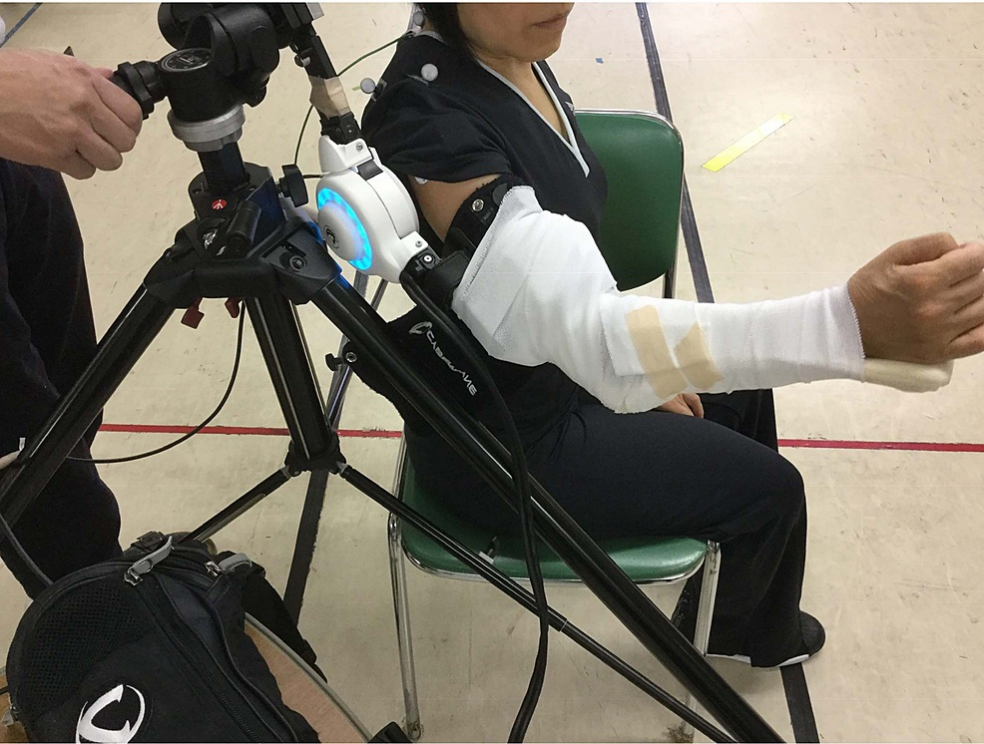
First hybrid assistive limb intervention.

The patient underwent a total of 10 HAL sessions of 20 minutes each for three months. It took approximately 20 minutes to prepare the electrodes and turn the HAL on and off. Training of the scapular flexion of the right shoulder with HAL lasted approximately 20 minutes, including rest periods. At least one physiatrist was present in case of an emergency, two assistants took the HAL suit on and off, and an engineer implemented motion analysis during shoulder training using HAL (Figure 2). The number of upper limb elevations per training session was adjusted based on the patient's fatigue level and condition. The elevation angle during training was set by the HAL side within the range of motion (ROM) not exceeding the range of motion of the joint in the seated position, which was confirmed by the physician each time the patient wore the HAL.

Clinical assessments were performed before, after, and six months after HAL intervention. A Trigno wireless electromyography (EMG) system (Delsys, Inc., Natick, MA, USA) was used to evaluate the muscle activation of the deltoid anterior, trapezius, infraspinatus, pectoralis major, triceps brachii, and biceps brachii muscles. Each muscle's activity was evaluated using EMG at 2000 Hz and filtered with a 30-400 Hz bandwidth filter using scripts on MATLAB 8.2 (MathWorks, Natick, MA, USA). The range of motion (ROM) of the joint during automatic and altruistic shoulder flexion was assessed to evaluate the shoulder joint function on the paralyzed side, and manual muscle test (MMT) was performed to evaluate the muscle strength of the shoulder flexion. The upper limb function on the paralyzed side was evaluated by grip strength, action research arm test (ARAT), and box and block test.

Results

The intervention period was 91 days, and the post-intervention period was 189 days. The average number of upper limb raising per training session was 142.7. No significant adverse events such as local shoulder pain or skin irritation were observed during the intervention and post-intervention periods.

**Figure 3:**
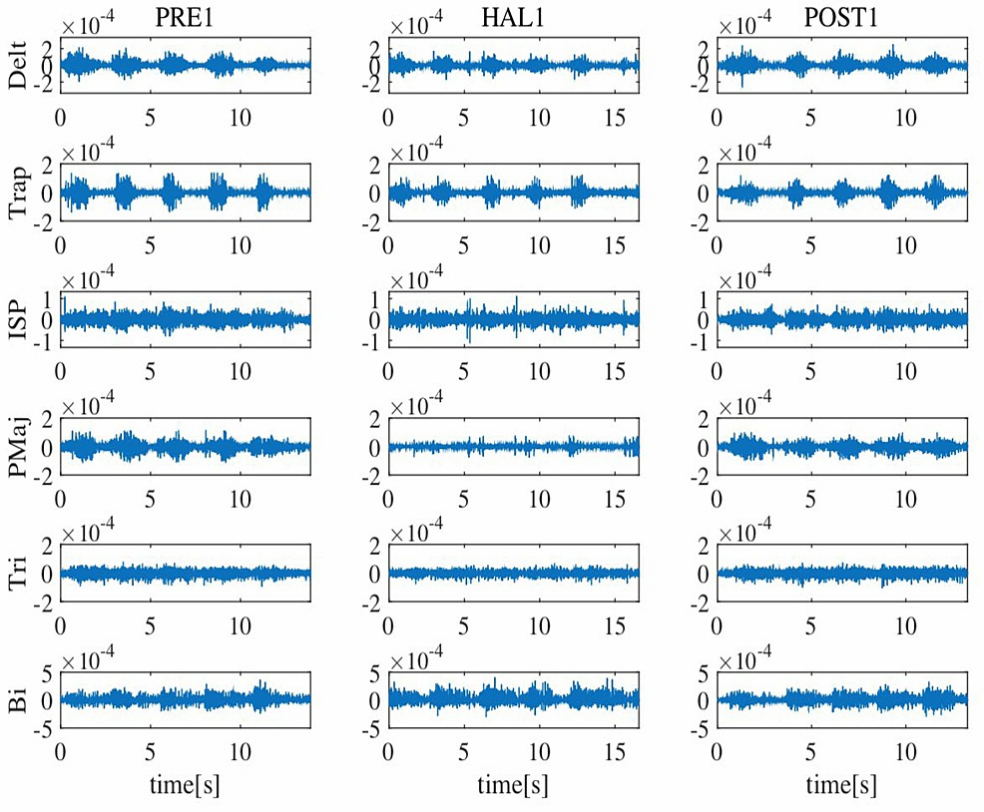
Muscle activities of the deltoid (Delt), trapezius (Trap), infraspinatus (ISP), pectoralis major (PMaj), triceps brachii (Tri), and biceps brachii (Bi) muscles before, during, and after the first hybrid assistive limb (HAL) session. Before the HAL intervention, PMaj is hyperactivated. During the HAL intervention, PMaj is less activated. PRE1: before the first HAL session; HAL: during the first HAL session, POST1: after the first HAL session.

**Figure 4:**
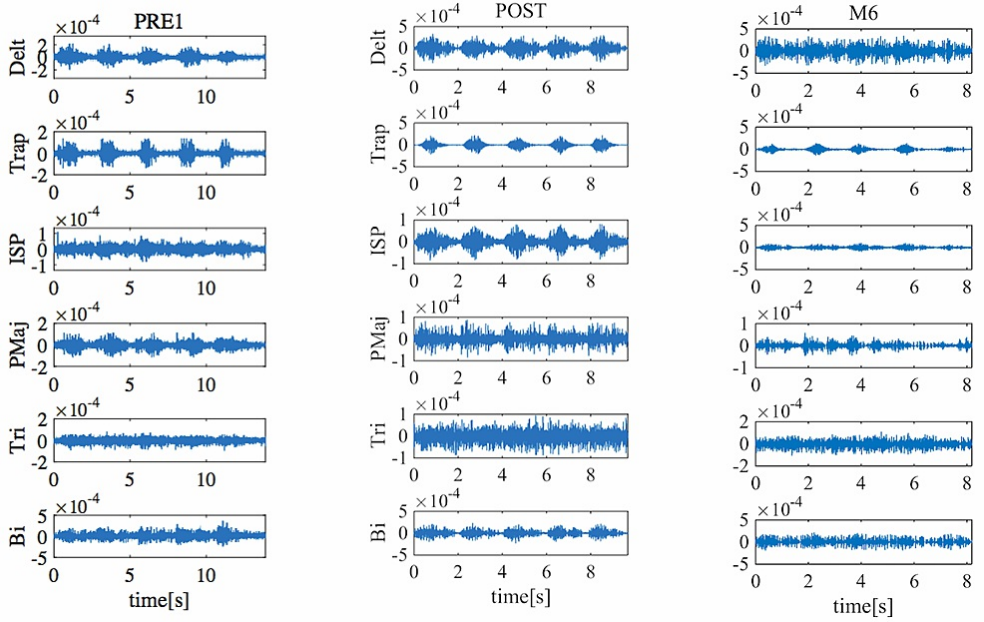
Muscle activities of the deltoid (Delt), trapezius (Trap), infraspinatus (ISP), pectoralis major (PMaj), triceps brachii (Tri), and biceps brachii (Bi) muscles one week before, one week after, and six months after hybrid assistive limb (HAL) session. Before the HAL intervention, PMaj is hyperactivated. During the HAL intervention, PMaj is less activated. PRE: one week before the HAL intervention; POST: one week after the HAL intervention; 6M: six months after the HAL intervention.

Figure 3 demonstrates the activities of the deltoid, trapezius, infraspinatus, pectoralis major, triceps brachii, and biceps brachii muscles before, during, and after the first HAL session. Co-activation was observed between the deltoid and pectoralis major muscles before the first session, but it decreased during and after the HAL session. Six months after the entire HAL intervention, the decrease of the co-activation between the deltoid and the pectoralis major has been maintained (Figure 4). Passive ROM changed from 105° to 115° during flexion, and active ROM changed from 65° to 105° during flexion (Table 1). The MMT grades of shoulder flexion changed from 2 to 4 (Table 1). The grip strength in the right hand before HAL was less than 5 kg, which means unmeasurable, but it increased to 7.4 kg after HAL (Table 1). In ARAT, the total score improved from 10 to 20 (Table 2). The box and block test in the right upper limb score increased from 1 to 8; moreover, it increased to 14 blocks six months after intervention (Table 2).

**Table 1:** Shoulder range of motion (ROM), shoulder manual muscle test (MMT), and grip strength. PRE: one week before hybrid assistive limb (HAL) intervention; POST: one week after HAL intervention; 6M: six months after HAL intervention.

		PRE	POST	6M
Shoulder flexion ROM	Passive	105	115	130
	Active	65	105	115
Shoulder MMT	Flexion	2	4	4
Grip strength	Right	0	7.4	6.4

**Table 2:** Fugl–Meyer assessment for upper extremity (FMA-UE), grip strength, action research arm test (ARAT), and box and block test (BBT). PRE: one week before hybrid assistive limb (HAL) intervention; POST: one week after HAL intervention; 6M: six months after HAL intervention.

		PRE	POST	6M
ARAT	Grasp	3	8	10
	Grip	3	5	6
	Pinch	0	1	1
	Gross movement	4	6	6
	Total	10	20	23
BBT	Right	1	8	14

The patient was visibly satisfied with the treatment and came to express her happiness by raising both hands. She was also able to turn over in her sleep and started to actively use it in her daily activities during the intervention. She started to actively perform daily voluntary training for the right upper limb for the first time after the onset of stroke.

After six months, a decrease in the co-activation of the pectoralis major muscle and other muscles while raising the upper limb on the paralyzed side was still observed. Although the grip strength of the paralyzed side slightly declined (from 7.4 kg at one week after HAL to 6.4 kg at six months after HAL), the active and passive shoulder ROM, shoulder flexion MMT, and the score of ARAT and BBT maintained or improved (Tables 1, 2).

## Discussion

This is the first case report of shoulder joint elevation training using a single-joint HAL for upper limb dysfunction after stroke.

The shoulder joint is capable of a wide range of movement, but due to its extensive mobility, it has an unstable structure. Because of the increased instability after stroke, shoulder ROM training on the paralyzed side is associated with a risk of trauma. Reports suggest that transitive ROM training should not be performed beyond 90° to prevent shoulder-hand syndrome, which is often a problem after stroke [5,16]. In this study, we performed an average of 142.7 automatic shoulder joint assistance exercises using the shoulder joint HAL for a total of 10 times in a patient with upper limb dysfunction after stroke, and we were able to perform the training without the appearance of shoulder joint pain. We believe that by setting the HAL and attachments in advance to avoid collision between the humerus and scapula during upper limb elevation, by raising the scapular plane and preventing the humerus from internally rotating, and by providing appropriate assistance using the HAL based on these settings, the same exercise could be safely repeated.

The surface electromyogram showed a decrease in the co-activation of the pectoralis major muscle and other muscles during the raising of the upper limb on the paralyzed side, suggesting that improvement in the coordination of the periarticular muscles of the shoulder joint may have resulted in an increase in muscle output. Our previous studies also showed the improvement of coordination in upper limbs after shoulder HAL intervention for a patient with C5 palsy [17] and elbow HAL for patients with spastic cerebral palsy [18].

The ROM of the shoulder flexion improved from 105° to 115° after the intervention. The active ROM of the shoulder flexion significantly improved from 65° to 105° after the intervention. In addition, the MMT of shoulder flexion also improved from 2 to 4. The results suggested that the patient became able to control the peri-shoulder muscles more easily because of the decrease in the co-activation of those muscles. The present study suggests that HAL may have had a motor learning effect on the shoulder joint.

Grip strength improved from less than 5 to 7.4 kg before the intervention. This result is consistent with another study result showing that proximal stabilization improves distal function [19]. The ARAT is a test that evaluates the ability to move the upper extremities through four categories, namely, grasping, griping, pinching, and gross movements, with a total of 19 items and a 57-point rating system. The minimal clinically important difference (MCID) in the life stage is estimated to be 5.7 points [19]. These results suggested that not only the shoulder joint function but also the entire upper limb function and movement may have been affected by the motor learning effect.

After six months, there was no decline in any item other than grip strength; in fact, the trend was toward improvement, indicating that we were able to provide effective training for the patient.

Since this is only a case report, further research is required to confirm the efficacy of this rehabilitation technique, but the results suggest the effectiveness of training using the shoulder HAL.

## Conclusions

We report the case of a 54-year-old female with right shoulder dysfunction due to hemiplegia after stroke who underwent right shoulder joint elevation training in a seated position using shoulder type HAL. We observed a decrease in co-activation between the deltoid and the pectoralis major muscles during upper limb elevation. Her right upper limb function, including passive and active range of motion of shoulder flexion, manual muscle test of shoulder flexion, and grip strength, improved. Moreover, the clinical test scores such as box and block test and action research arm test increased, and she started to use her right upper limb in her daily activities. No adverse events, including shoulder pain, were seen. Therefore, shoulder HAL may be an effective rehabilitation for upper limb dysfunction after stroke.
